# Update on Robotic Laparoscopic Radical Prostatectomy

**DOI:** 10.1100/tsw.2006.394

**Published:** 2006-02-17

**Authors:** Garrett S. Matsunaga, Thomas E. Ahlering, Douglas W. Skarecky

**Affiliations:** Division of Urological Oncology, University of California, Irvine Medical Center, Department of Urology, Orange, CA 92868, USA

**Keywords:** robot, prostatectomy, laparoscopic, prostate, cancer, review

## Abstract

The da Vinci surgical robot has been shown to help shorten the learning curve for laparoscopic radical prostatectomy (LRP) for both laparoscopically skilled and naïve surgeons[1,2]. This approach has shown equal or superior outcomes to conventional laparoscopic prostatectomy with regard to ease of learning, initial complication rates, conversion to open, blood loss, complications, continence, potency, and margin rates. Although the data are immature to compare oncologic and functional outcomes to open prostatectomy, preliminary data are promising.Herein, we review the technique and outcomes of robotic-assisted laparoscopic radical prostatectomy (RALP).

## INTRODUCTION

Since the first laparoscopic nephrectomy was performed by Clayman et al. in 1991[3], minimally invasive surgical techniques have gradually permeated the realm of urologic operations. Although laparoscopic techniques were initially limited to extirpative procedures, more complex reconstructive cases are now being performed. Laparoscopic radical prostatectomy (LRP) is arguably one of the most difficult urologic laparoscopic operations, as it requires both extirpative and reconstructive laparoscopic skill sets.

In 1992, Schuessler et al. reported their experience of nine laparoscopic radical prostatectomies[4]. With limited laparoscopic instruments, average operative time was 9.4 h with more than half of the time devoted to the urethrovesical anastamosis. No clear advantage over open radical prostatectomy with regard to length of hospitalization, convalescence, continence, or potency was found. In fact, they concluded that advances in instrumentation and technology would likely be required to show any possible advantage to this procedure over the open approach.

The adoption of LRP as a feasible approach to prostate cancer required the tenacity and commitment of two separate groups in Paris. Guillonneau and Vallancien[5], using a transperitoneal approach, reported impressive reductions in operative time to an average of <4 h. Similarly, Abbou et al.[6] reported median operating times of 4.3 h without and 5.1 h with pelvic lymphadenectomy in 33 patients after 10 procedures had been performed to standardize their technique. These surgeons were skilled laparoscopists and made a commitment to perform a sufficient number of cases before deciding on the feasibility of LRP. The latter factor was crucial to success because the “learning curve” was found to be in excess of four times the original series reported by Schuessler et al.

Despite significant advances in technique and instrumentation, standard laparoscopy has inherent limitations. Due to the rigidity of the instrument shaft and the fixed position of the trocar on the abdominal wall, the degrees of freedom of movement are limited to four. Another drawback is the two-dimensional view of the camera system, which makes it difficult to determine spatial distance. In addition, all movements are counterintuitive, and control of the endoscope remains in the hands of an assistant or a voice-controlled robotic arm.

With the advent of robotic laparoscopic surgery using a master-slave system, some of the drawbacks of standard laparoscopy can now be overcome. Originally developed for use in battlefield open trauma surgery, with the surgeon controlling the surgical manipulators from a safe distance (i.e., telepresence surgery)[7], such systems have now been adapted for medical use in the civilian world and have already been proven to be effective in cardiac surgery as well as other procedures[8,9,10].

The da Vinci robotic surgical system (Fig. 1) is a master-slave robotic system consisting of a free-standing robotic tower and a surgeon console. The robotic tower has a camera arm and two or three instrument arms. The unique video system provides 10× magnification and three-dimensional image of the surgical field. The surgeon's console is an ergonomically designed interface for controlling the Endowrist™ articulated instruments with 6 degrees of freedom: in, out, left, right, up, down, rotational axis, pitch, and yaw. The bidirectional articulation, plus grip, mimic the actual hand and wrist movements of the surgeon. In addition, this system has tremor control and 1:5 motion scaling. The surgeon's hand-eye axis is positioned in such a manner that the illusion of operating directly on the patient is created[11]. Although there is no tactile (haptic) feedback, the surgeon quickly learns to rely on visual cues for determining tissue and suture tension.

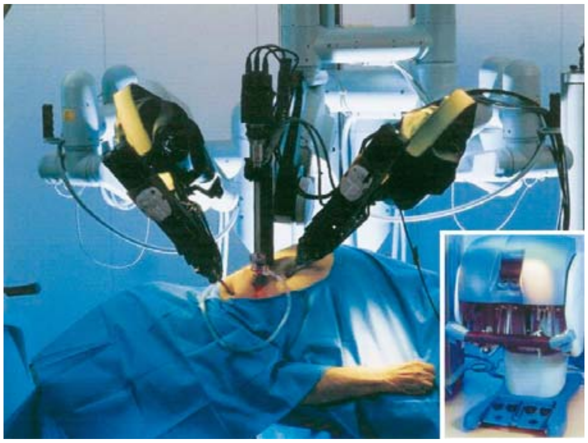

**FIGURE 1.** The da Vinci surgical system (Intuitive Surgical, Sunnyvale, CA) consists of a central arm that controls the laparoscope and camera as well as two side arms that are used to control a variety of grasping and cutting instruments. The surgeon can activate either the two side arms to direct the instruments or the central arm to adjust the surgical view. (Figure reprinted from *Surgical Endoscopy* **18(12),** 1694–1711, 2004. With kind permission of Springer Science and Business Media.)

During robotic-assisted laparoscopic radical prostatectomy (RALP), the assistant is situated at the patient's side, while the primary surgeon sits and operates at the console. The assistant is responsible for changing instruments for the robot manipulators and cleaning the lens of the laparoscope and replacing it in the third robot arm. The assistant's other duties also include retracting, suctioning, and passing sutures via the other ports.

## TECHNIQUE

The technique of LRP varies from center to center. Removal of the prostate gland can be performed in an ascending (from apex to base) or descending fashion (from base to apex). In most of the reported cases, an intraperitoneal approach has been used (e.g., Institut Mutualiste Montsouris, Paris, France; Klinikum Heilbronn, Heilbronn, Germany; Vattikutti Institute, Detroit, MI; and University of California at Irvine, Orange, CA). However, several centers have also described the use of an extraperitoneal approach (e.g., Erasme Hospital, Brussels, Belgium and University of Leipzig, Leipzig, Germany) (Fig. 2).

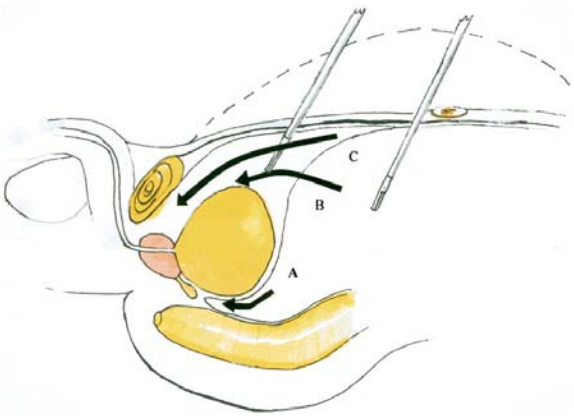

**FIGURE 2.** Initial surgical approaches to LRP. **A:** Montsouris technique. **B:** Heilbronn, Vattikuti Institute, University of California Irvine technique. (Note: Heilbronn uses an ascending prostatic dissection, whereas Vattikutti Institute and University of California Irvine use a descending dissection). **C:** Extraperitoneal technique. (Figure reprinted from *Surgical Endoscopy* **18(12),** 1694–1711, 2004. With kind permission of Springer Science and Business Media.)

The surgeons who have performed the greatest number of robot-assisted prostatectomies use the intraperitoneal descending method described by the group in Montsouris, but they modify the technique by doing the retrovesical portion at the end, rather than at the beginning of the procedure[12,13]. Some surgeons have used an extraperitoneal technique; however, the series reported thus far have been very small[14,15,16].

## University of California, Irvine Technique

After insufflation of the peritoneal cavity, a 12-mm trocar for the camera is placed at the umbilicus. The remaining ports are then inserted under laparoscopic guidance. Two nondisposable robotic 8-mm trocars are placed pararectally for the robotic instrument arms. A 12-mm trocar off the right anterior superior iliac crest and a 5-mm trocar in the right upper quadrant are placed for the laparoscopic assistant. If the fourth robotic arm is available, a third robotic 8-mm trocar is placed in the left lower quadrant, lateral to the left robotic arm port, and just off the left anterior superior iliac crest (Fig. 3).

Initially, the 0-degree laparoscope is used with Maryland bipolar forceps in the left robotic arm and the monopolar scissors in the right hand. An inverted “U” incision in the peritoneum is made from one inguinal ring to the umbilicus then to the contralateral inguinal ring. In this fashion, the space of Retzius is entered and the bladder is “dropped” from the anterior abdominal wall and the pelvic side wall exposed. The fat over the puboprostatic ligaments, prostate, and endopelvic fascia is then removed to improve visualization in steps of dissection to follow.

Next, the endopelvic fascia is incised, exposing the levator ani muscles. The superficial dorsal veins are coagulated with bipolar cautery and transected, the puboprostatic ligaments are divided, and the pelvic musculature is completely separated from the dorsal venous complex and prostatic apex.

Using a 45-mm Endo-GIA stapler (Ethicon Endosurgery, Cincinnati, OH or U.S. Surgical Inc., Norwalk, CT), the deep dorsal venous complex is stapled and transected. It is important to maintain the stapler on the dorsal vein for at least a minute before activating the cutting blade to achieve appropriate hemostasis.

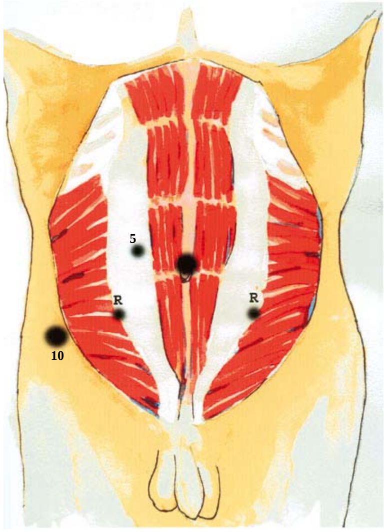

**FIGURE 3.** Port placements for the robotic technique used at the University of California at Irvine. (Figure reprinted from *Surgical Endoscopy* **18(12),** 1694–1711, 2004. With kind permission of Springer Science and Business Media.)

The 30-degree down laparoscope is now utilized during the development of the plane between the prostate and bladder. As a rule, the perivesicle fat is adherent to the bladder, and the line of demarcation between the bladder and prostate is where the extravesical fat ends. Another method of defining the prostatovesicle junction is by gently grasping and tugging at the anterior bladder wall. The bladder will easily move with traction, whereas the prostate will remain fixed. Electrocautery is used to divide the anterior bladder neck, exposing the Foley catheter. The balloon on the Foley catheter is then deflated and the tip of the catheter is delivered from the bladder. With the assistant pulling the tip of the Foley catheter anteriorly toward the abdominal wall, the bladder neck is dismembered from the prostate using a bladder neck–preserving technique. Posteriorly, the seemingly thick anterior layer of Denonvillier’s fascia is encountered and incised transversely to gain access to the vas deferens and seminal vesicles.

The vas deferens are identified and dissected proximally to the tip of the seminal vesicles before being transected. A portion of each vas is then excised and the remaining stumps are used to hold cephalic and anterior traction on the prostate. The seminal vesicles are dissected free with bipolar and sharp dissection. The posterior layer of Denonvillier’s fascia is then incised and the plane between the posterior prostate and rectum is mobilized. This step is done carefully without the use of monopolar electrocautery to reduce the risk of rectal injury.

Holding the vasa and seminal vesicles anteriorly, the prostatic lateral vascular pedicles are placed on stretch and thus are easily identified. Laparoscopic bulldog clamps (30 mm) are placed on the vascular pedicles prior to transection with scissors close to the prostate. Again, no thermal energy is used during the preservation of the neurovascular bundles. Posterolateral dissection is continued toward the apex of the prostate carefully releasing and preserving the neurovascular bundles. If the nerves are being excised, the bulldog clamps are not used. Instead the pedicles are cauterized with the bipolar cautery then transected. The neurovascular bundles are excised off the perirectal fat using bipolar and sharp dissection.

The urethra is then transected with the scissors just distal to the prostatic apex. The prostate is then placed in a laparoscopic entrapment sac and positioned in the paracolic gutter. It is delivered out of the abdomen at the very end of the procedure by enlarging the umbilical port as needed.

If bulldog clamps were utilized, 6-in. 3-0 polyglactin sutures on an RB needle are then used to oversew the lateral pedicles for hemostasis. A locking, figure-of-eight stitch is placed through a part of the pedicle. The bulldog clamp is then released and all bleeding vessels in the pedicle are then precisely identified and oversewn.

A van Velthoven running stitch technique is used to perform the urethrovesical anastamosis. Two 6-in. monofilamentous absorbable sutures (3-0 poliglecaprone on an SH needle) are tied together at the ends with a small piece of the patient's vas deferens used as a pleget. To assist in differentiating one suture from the other, one of the two sutures is dyed blue. Each needle is respectively passed outside-in at the 5:30 and 6:30 position of the bladder, so that the preformed knot lies on the outside of the bladder. The 0-degree laparoscope is now replaced. The needles are then passed inside-out on the corresponding area of the urethra, and a running stitch is continued on both sides of the urethra and bladder neck (Fig. 4). At the 10 o'clock position, a transition stitch is performed such that the suture now traverses outside-in on the urethra and inside-out on the bladder. This technique leaves only one intracorporeal knot to be performed, which is at the 12 o'clock position where both running sutures meet. The resulting knot bridges across the anastamosis.

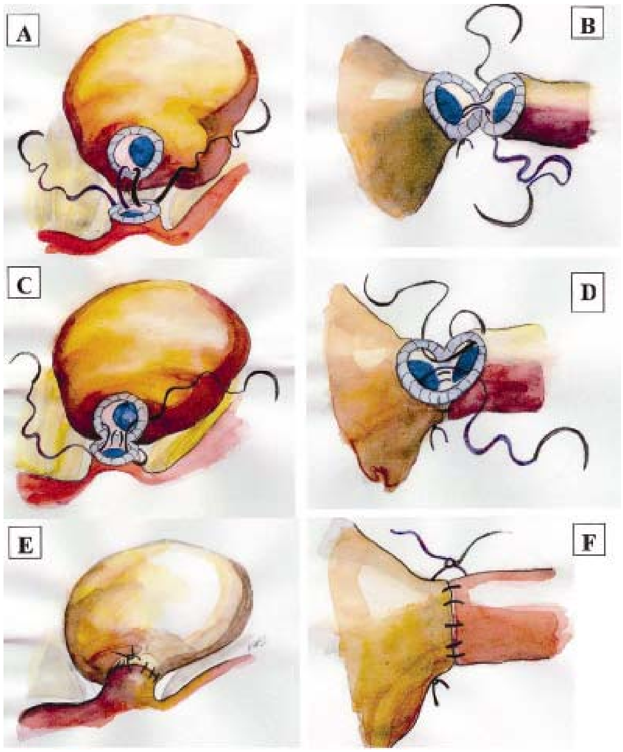

**FIGURE 4**. Single-knot method for laparoscopic running urethrovesical anastomosis. (Reprinted from Urology 61(4), 699–702, 2003. With kind permission from Elsevier.)

A new 18F silastic foley catheter is placed into the bladder and the bladder is irrigated to check for any leaks. No drain is routinely placed. The robot is undocked and the prostate is brought out through the umbilical port.

If a lymph node dissection is performed, this is done just prior to the vesicourethral anastamosis. With the prostate removed, there is excellent exposure of the iliac vessels. Maryland bipolars and monopolar scissors are used for the dissection. The limits of the dissection are the same as for the open technique: external iliac vein superiorly, lymph node of Cloquet distally, pelvic side wall laterally, obturator nerve inferiorly, and origin of the hypogastric artery proximally.

## PROGRAM ESTABLISHMENT — LEARNING CURVE

As for any new procedure or technique, there is a learning curve for LRP. The operative time for the first reported series of LRP averaged 9.4 h; however, centers with extensive experience have now reported operative times of 3.3–4.4 h[11]. The learning curve for standard laparoscopic prostatectomy is estimated to be 40 cases, based on operative time. In their analysis of their first 120 patients, Guillonneau and

Vallancien[5] reported an average operative time of 4.7 h in their initial 40 patients, improving to 4.1 and 3.8 h, respectively, in subsequent groups of 40 patients, with an average of 3.95 h for the last 80 patients.

When robot-assisted laparoscopic prostatectomy is done by experienced open surgeons, the learning curve appears to be accelerated. Remarkably similar results in both academic (Menon et al.[17] and Ahlering et al.[18]) and community (Patel et al.[19]) settings showed that robotic assistance enabled both laparoscopically skilled (Patel) and naïve (Menon and Ahlering) surgeons to achieve operative times similar to an accomplished standard laparoscopist within 18, 10, and 20 cases, respectively (Fig. 5).

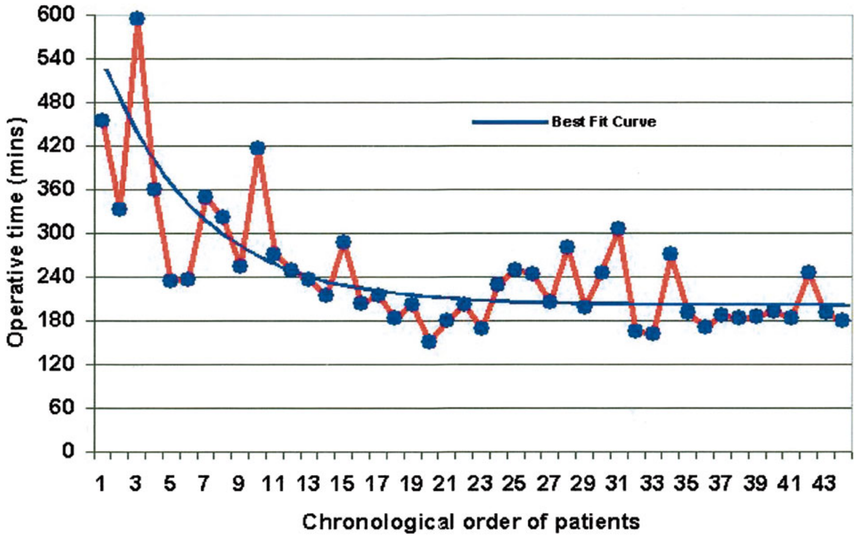

**FIGURE 5.** Operative times for robotic-assisted radical prostatectomy at the University of California at Irvine. (Figure reprinted from *Surgical Endoscopy* **18(12),** 1694–1711, 2004. With kind permission of Springer Science and Business Media.)

## PERIOPERATIVE RESULTS

Results of all reported RALPs were recently summarized very nicely by El-Hakim and Tewari[20]. The average patient was 61 years of age, PSA 7.6, BMI of 27.3, and prostate volume of 53.8 g; 47.5% were Gleason score 6 or less, 35.7% were Gleason score 7, and 12.2% were Gleason score 8 or higher. Mean operative time was 222 min and estimated blood loss 231 cc. Only 1.1% of cases were converted to open and blood transfusion rate was 0.3%. Average hospital stay was 1.4 days and catheters were removed at 8.1 days. Minor complications occurred in 4.55% of cases, including UTI, anastomotic leaks, ileus, and port site bleeding. Major complications occurred in 3.75%, including deep venous thrombosis, pulmonary emboli, obturator nerve injury, anastomotic disruption, delayed bleeding, and wound dehiscence.

As these series come from academic institutions, some question whether these results would translate into a community setting. Recently, a large community series of 200 consecutive RALP patients was reported by Patel and associates[19]. Baseline patient characteristics were essentially similar to other reported series. Likewise, operative times (141.2 min), EBL (75.1 cc), transfusion rates (0%), conversions (0%), hospital stay (1.1 days), and length of catherization (7.9 days) were similar to large academic institutions. Complication rate was 2%, including rectal injuries (2), pelvic hematoma (1), and bladder neck contracture (1) (see Table 1).

**Table 1**
**Outcomes of Reported RALP Series**

| First Authors | No. of Patients | Age (Years) | Method | OR Time (h) | EBL (ml) | Tx Rate (%) | Anastomosis | Open Conversion Rate (%) | Margin-Positive Rate (%) | | | | | |
|---|---|---|---|---|---|---|---|---|---|---|---|---|---|---|
| | | | | | | | | | Overall | pT2 | pT3a | pT3b | pT4 | Gl <7/=>7 |
| Rassweiler [27] | 33 | 68 | I, A | 7.5[a] | na | na | Interrupted | 0 | 18 | na | na | na | na | na |
| Pasticier [28] | 5 | 58 | I, D | 3.7 | 800 | na | Interrupted | 0 | 20 | 33 | na | na | na | na |
| Menon[2] | 40 | 60.7 | I, D | 4.6 | 391 | 0 | Interrupted | 2.5 | 17.5 | na | na | na | na | na |
| Tewari[29] | 100 | na | I, D | na | na | na | Interrupted | na | na | na | na | na | na | na |
| Menon[17] | 100 | 60 | I, D | 3.3 | 149 | 0 | Running | na | 15 | 10.6 | 40 | na | na | 14/16 |
| Ahlering[18] | 60 | 62.9 | I, D | 3.9[b] | 103 | 0 | Running | 0 | 16.7 | 4.5 | 47 | na | 100 | na |
| Patel[19] | 200 | 59.5 | I, D | 2.35 | 75.1 | 0 | Running | na | 10.5 | 5.7 | 28.5 | 20 | 33 | na |

| First Authors | Mean Follow- Up (Months) | Hospital Stay (Days) | Catheter Use (Days) | Time of Postoperative Assessment (Months) | Continence (%) | Criteria | Erectile Function (bns/uns) (%) | Complication Total, Major/Minor (%) |
|---|---|---|---|---|---|---|---|---|
| Rassweiler[27] | na | na | 6.8 | na | na | na | na | na |
| Pasticier [28] | na | na | 5.5 | na | na | na | na | 0 |
| Menon[2] | 6.5 | na | na | 1.5 | na | na | 29/na | 15, 0/15 |
| Tewari[29] | na | na | na | 6 | na | na | 58* | na |
| Menon[17] | 5.5 | 0.96§ | 4.2 | 6 | 92 | 0 or pad use for protect-tion only | na | 8 |
| Ahlering [18] | na | 1.1 | 7 | 3 | 75 | 0 pads | na | 6.7 |
| Patel[19] | 9.7 | 1.1 | 7.9 | 1,3,6,9,12 | 47,78,89,92,98 | 0 pads | na | 2 |

I, intraperitoneal; A, ascending; D, descending; OR time, operative time; Tx rate, transfusion rate; EBL, estimated blood loss; na, not available; PSA < 0.2 ng/ml (prostate specific antigen); bns, bilateral nerve sparing; uns, unilateral nerve sparing.

* Assessed with validated patient questionnaire.

§ Length of hospital stay for 95/100 patients.

[a] Includes pelvic lymph node dissection in 21/33 patients.
[b] Excludes robotic setup.

## ONCOLOGICAL CONTROL

The primary goal of radical prostatectomy is to attain a surgical cure. One surrogate in achieving this goal can be measured by the margin status of the specimen and the absence of chemical or clinical recurrence. The overall margin-positive rate in reported series of RALP averages 15% with a range of 10.5–20%. However, in pathologically organ-confined disease (pT2), the margin positive rate is lower, ranging from 4.5–16.7%.

An important aspect of radical prostatectomy is the reduction of iatrogenic-positive margins in otherwise organ-confined prostate cancer. Recently, Ahlering et al.[21] reported several technical points that further aid in apical dissection and thereby minimize the risk of positive surgical margins (removal of all of the fat overlying the dorsal venous complex (DVC) and prostate, division of the puboprostatic ligaments and dissection of the levator fibers to expose and increase the DVC length, and division of the DVC using a laparoscopic vascular stapler). Using this technique, the authors reduced their overall positive margin rates from 36–16.7% and pT2-positive margin rate from 27.3–4.7%. The transferability of this technique has been demonstrated now in Rochester, NY with very similar rates (Eichel and partner, personal communication).

## CONTINENCE

Evaluation of continence rates between reported series can be difficult due to the subjectivity of the surgeon and patients, the use of different or nonvalidated questionnaires by surgeons, and variability in the definition of continence by surgeons and patients. With ≥6 months follow-up, the urinary continence rates in reported RALP series range from 85–98%. However, to assess this parameter truly, the use of a validated continence questionnaire is essential; data accumulated by any other instruments are suspect. In Patel's[19] community series of 200 patients, 27% were continent (no pads) immediately after catheter removal, 47% at 1 month, 82% at 3 months, 89% at 6 months, 92% at 9 months, and 98% at 1 year.

Our experience of 185 men finds that at 1 week, 33% are pad free. At 1 month, 50% are pad free and 25% use a security pad. At 3 months, 80% of patients use no pads and 15% use a security or one pad per day, and 5% use >1 pad per day. The overall rate of pad-free continence was 85% at 6 months, 92% at 1 year.

In addition to technical factors, there seem to be characteristics of patients that can influence postoperative results. We recently prospectively reviewed 100 men undergoing RALP; 19 men were obese (BMI > 30) and 81 were nonobese (BMI < 30). The two groups had similar need for transfusion, length of stay, and pathological outcomes. Obese patients had significantly worse baseline urinary and sexual function, suffered more complications, recovered urinary function more slowly, and also demonstrated a strong trend for delay in recovery time[22].

## SEXUAL FUNCTION

Preservation of sexual function has a significant impact on the quality of life of men who undergo radical prostatectomy. Wide variability in the use/lack of use of validated questionnaires (e.g., IIEF International Index of Erectile Function) hinders the evaluation of sexual function after RALP. The use of validated questionnaires is essential to the acquisition of accurate data that can then be used to correlate erectile function with operative technique. In addition, rates of preservation of sexual function can be affected by patients' age, whether one or both neurovascular bundles were preserved, and whether the patient was sexually active prior to surgery. It is important to distinguish after the procedure between patients who are naturally potent and those who are potent only with medications.

El-Hakim and Tewari[20] summarized the available series on postoperative sexual function in RLP. At an average of 7.7 months, 49.5% of patients were having intercourse and 79% had return of erections, with or without medical assistance.

Menon reports identifying a lattice of nerves on the ventral and lateral prostatic fascia as "The veil of Aphrodite"[23]. They hypothesize that preservation of the lateral prostatic fascia avoids transaction of these accessory nerves and decreases the risk of traction injury to the major neurovascular bundle during this dissection. In a selected subset of 35 subjects, a rate of 95% at 1 year is reported for erections strong enough for intercourse.

The mainstay to nerve preservation is avoidance of nerve transsection followed by reduction of traumatic injury. Techniques such as bipolar electrocautery, harmonic scalpel, and ligasure have been introduced in an attempt to reduce thermal injury and by inference decreased stray electrical energy. Using a dog model, Ong et al.[24] demonstrated the potential for significantly decreased erectile response by traumatic injury by monopolar and bipolar hemostatic cautery used in close proximity of the neurovascular bundles (NVB). A feasibility study by Ahlering et al.[25] describes the cautery free, clip-free, dissection of the cavernous nerves to be safe to perform in RALP. Our current technique involves placing bulldog clamps on the lateral pedicles prior to cautery-free, sharp dissection of the pedicles and the neurovascular bundles off the prostate.

Although complete information regarding potency will require at least 2 years of follow-up, we have already experienced dramatic improvement over our previous technique using bipolar to control the vascular pedicle; 43 vs. 8% of men (≤65 years and preoperative IIEF-5 of 22–25) have return of erectile function with the cautery-free technique at 3 months with or without PDE-5 inhibitors. Additionally, only 18% with the cautery-free technique did not have partial erections compared to 68% in bipolar group ($p = 0.01$)[26].

## ECONOMIC

Large initial startup costs have been a limitation to widespread purchase and integration of robotics at many centers. Currently, the cost of the da Vinci robot is $1.2 million with a $100,000 annual maintenance contract. In addition, there is the cost for use of the reposable robotic instruments. The instruments vary in the number of times they may be reused, however, the average cost per case is about $200–300 per instrument.

A recent cost comparison of open retropubic prostectomy (RRP), laparoscopic, and robotic was reported by Lotan and associates[30]. Hospital costs (room and board, i.v. fluids, medication, and blood transfusions), operating room costs (OR time and equipment), and surgeon's fee were analyzed. With regard to the da Vinci robot, cost was estimated at $1.2 million with a $100,000 annual maintenance fee with assumed annual case load of 300 cases for a period of 7 years. RRP was the most cost-effective approach ($5,554) with a cost advantage over LRP and RALP of $487 and $1,726, respectively. Even when the initial $1.2 million cost of the robot was excluded, RALP was still $1,155 more costly. Increasing the average case load to 500 cases per year would only result in about a $350 dollar decrease in cost per case.

Mean OR times used for RRP, LRP, and RALP were 160, 200, and 140 min, respectively. Mean length of stay for RRP, LRP, and RALP was 2.5, 1.3, and 1.2 days. Despite the reduction in OR times and hospital stays, it was not sufficient to offset the increased costs of equipment used in the laparoscopic and robotic approaches. There was no point at which RALP could meet equivalence to RRP. If the initial robot cost was excluded, robotic instrument costs would need to be less than $550 ($1,150 decrease) to meet cost equivalence with RRP. Alternately, a robot price of $500,000, annual maintenance fee of $34,000/year, and total instrument cost of $500 per case would be required to achieve cost equivalence with RRP[30].

The authors recognized that the value in earlier return to work was not specifically examined in their study. Other authors have noted a 17–25 day earlier return to full convalescence with LRP compared to RRP. Based on U.S. Census Bureau information, an average American would earn $2,115 in a 15-day period. This would then make LRP and RALP more cost effective than the open technique[30]. Studies looking at earlier return to work would need to be evaluated to confirm this.

Lastly, as with all new technology, as time passes, there may be reductions in production costs in the robot and its equipment which will make RLP less costly.

## CONCLUSION

Early results of patients undergoing RALP appear to be at least equal to LRP or open radical prostatectomy. Long-term oncologic and functional outcomes will need to be followed to confirm RALP as the new gold standard.

## ACKNOWLEDGMENTS

Figs. 1, 2, 3, and 5 with kind permission of Springer Science and Business Media. Permission free of charge does not prejudice any rights we might have to charge for reproduction of our copyrighted material in the future.

## References

1. Ahlering, T., Skarecky, D., Lee, D., and Clayman, R. (2003) Successful transfer of open surgical skills to a laparoscopic environment using a robotic interface: initial experience with laparoscopic radical prostatectomy. *J. Urol*. **169**, 1649. [Abstract]
2. Menon, M., Shrivastava, A., Tewari, A., Sarle, R., Hemal, A., Peabody, J., and Vallancien, G. (2002) Laparoscopic and robot assisted radical prostatectomy: establishment of a structured program and preliminary analysis of outcomes. *J. Urol*. **168,** 945–949.
3. Clayman, R., Kavoussi, L., Soper, N., Dierks, S., Meretyk, S., Darcy, M., Roemer, F., Pingleton, E., Thomson, P., and Long, S. (1991) Laparoscopic nephrectomy: initial case report. *J. Urol*. **146**, 278–282.
4. Schuessler, W., Schulam, P., Clayman, R., and Vancaille, T. (1992) Laparoscopic radical prostatectomy: initial case report. *J. Urol*. **147**, 246.
5. Guillonneau, B. and Vallancien, G. (2000) Laparoscopic radical prostatectomy: the Montsouris experience. *J. Urol*. **163**, 418–422.
6. Abbou, C.C., Salomon, L., Hoznek, A., Antiphon, P., Cicco, A., Saint, F., Alame, W., Bellot, J., and Chopin, D.K. (2000) Laparoscopic radical prostatectomy: preliminary results. *Urology* **55,** 630–634.
7. Green, P., Piantanida, T., Hill, J., et al. (1991) Telepresence: dexterous procedures in a virtual operating field. *Am. Surg*. **57,** 192. [Abstract]
8. Cardiere, G., Himpens, J., Vertruyen, M., and Favretti, F. (1999) The world's first obesity surgery performed by a surgeon at a distance. *Obes. Surg*. **9**, 206–209.
9. Shennib, H., Bastawisy, A., McLoughlin, J., and Moll, F. (1999) Robotic computer-assisted telemanipulation enhances coronary artery bypass. *J. Thorac. Cardiovasc. Surg*. **117,** 310–313.
10. Young, J., Chapman, W., Kim, V., Albrecht, R., Ng, P., Nifong, L., and Chitwood, W. (2002) Robotic-assisted adrenalectomy for adrenal incidentaloma: case and review of the technique. *Surg. Laparosc. Endosc. Percutan. Tech.* **12**, 126–130.
11. Basillote, J., Ahlering, T., Skarecky, D., Lee, D., and Clayman, R. (2004) Laparoscopic radical prostatectomy: review and assessment of an emerging technique. *Surg. Endosc*. **18**, 1694–1711.
12. Perer, E., Lee, D., Ahlering, T., and Clayman, R. (2004) Robotic revelation: laparoscopic radical prostatectomy by a non-laparoscopic surgeon. *J. Am. Coll. Surg*. **197,** 693–696.
13. Tewari, A., Peabody, J., Sarle, R., Balakrishnan, G., Hemal, A., Shrivastava, A., and Menon, M. (2002) Technique of da Vinci robot-assisted anatomic radical prostatectomy. *Urology* **60,** 569–572.
14. Antiphon, P., Hoznek, A., Gettman, M., la Taille, A., Salomon, L., Katz, R., Borkowski, T., and Abbou, C. (2003) Extraperitoneal laparoscopic robot assisted radical prostatectomy. *J. Urol*. **169**, Abstr V965.
15. Dakwar, G., Ahmed, M., Sawczuk, I., Rosen, J., Lanteri, V., and Esposito, M., (2003) Extraperitoneal robotic prostatectomy: comparison of technique and results at one institution. *J. Urol.* **169**, 1660. [Abstract]
16. Gettman, M., Hoznek, A., Salomon, L., Katz, R., Borkowski, T., Antiphon, P., Lobontiu, A., and Abbou, C. (2003) Laparoscopic radical prostatectomy: description of the extraperitoneal approach using the da Vinci robotic system. *J. Urol*. **170**, 416–419.
17. Menon, M., Shrivastava, A., Sarle, R., Hemal, A., and Tewari, A. (2003) Vattikuti Institute prostatectomy: a single-team experience of 100 cases. *J. Endourol*. **17,** 785–790.
18. Ahlering, T., Woo, D., Eichel, L., Lee, D., Edwards, R., and Skarecky, D. (2004) Robot-assisted versus open prostatectomy: a comparison of one surgeon's outcomes. *Urology* **63**, 819–822.
19. Patel, V., Tully, A., Holmes, R., and Lindsay, J. (2005) Robotic radical prostatectomy in the community setting – the learning curve and beyond: initial 200 cases. *J. Urol*. **174,** 269–272.
20. El-Hakim, A. and Tewari, A. (2004) Robotic prostatectomy – a review. *MedGenMed* (online) **6(4),** 20.
21. Ahlering, T., Eichel, L., Edwards, R., Lee, D., and Skarecky, D. (2004) Robotic radical prostatectomy: a technique to reduce pT2 positive margins. *Urology* **64**, 1224–1228.

22. Ahlering, T., Eichel, L., Edwards, R., and Skarecky, D. (2005) Impact of obesity on clinical outcomes in robotic prostatectomy. *Urology* **65**, 740–744.
23. Menon, M., Kaul, S., Bhandari, A., Shrivastava, A., and Hemal, A. (2005) Prospective non-randomized comparison of standard nerve sparing and the "Veil of Aphrodite": analysis of post operative potency using the International Index of Erectile Function-5 questionnaire. *J. Urol*. **173**, 270 Abstr 999.
24. Ong, A., Su, L., Varkarakis, I., Inagaki, T., Link, R., Bhayani, S., Patriciu, A., Crain, B., and Walsh, P. (2004) Nerve sparing radical prostatectomy: effects of hemostatic energy sources on the recovery of cavernous nerve function in a canine model. *J. Urol*. **172**, 1318–1322.
25. Ahlering, T., Eichel, L., Chou, D., and Skarecky, D. (2005) Feasibility study for robotic radical prostatectomy cautery-free neurovascular bundle preservation. *Urology* **65**, 994–997.
26. Ahlering, T., Eichel, L., and Skarecky, D. (2005) Early potency outcomes with cautery-free neurovascular bundle preservation with robotic laparoscopic radical prostatectomy. *J. Endourol*. **19,** 715–718.
27. Rassweiler, J., Binder, J., and Frede, T. (2001) Robotic and telesurgery: will they change our future? *Curr. Opin. Urol*. **11**, 309–320.
28. Pasticier, G., Rietbergen, J., Guillonneau, B., Fromont, G., Menon, M., and Vallancien, G. (2001) Robotically assisted laparoscopic radical prostatectomy: feasibility study in men. *Eur. Urol*. **40,** 70–74.
29. Tewari, A., Dasari, R., Hasan, M., Hemal, A., Peabody, J., Sarle, R., Bansal, A., and Menon, M. (2003) Sexual function following robotic prostatectomy: a study using expanded prostate cancer index composite (EPIC) quality of life instrument. *J. Urol*. **169**, Abstr 1615.
30. Lotan, Y., Cadeddu, J., and Gettman, M. (2004) The new economics of open, laparoscopic and robot assisted techniques. *J. Urol*. **172,** 1431–1435.

## BIOSKETCHES

**Thomas Ahlering, M.D.,** is Professor and Chief of the Division of Urologic Oncology at the University of California, Irvine. Now in its 4th year of robot-assisted surgery, the UC, Irvine robotic-assisted laparoscopic prostatectomy experience is one of the oldest programs in the world. Dr. Ahlering initiated the program and has performed minimally invasive robotic prostatectomies on more than 350 patients and is a recipient of Intuitive Surgical's Pioneer of da Vinci Urology Surgery (2005).

**Garrett Matsunaga, M.D.,** is a Clinical Instructor and Robotic Surgical Fellow in the Department of Urology at the University of California, Irvine, and an assistant surgeon in robotic prostatectomy.

**Douglas Skarecky, B.S**. is a Staff Research Assistant in the Department of Urology at the University of California, Irvine, and has published more than a dozen articles on robotic prostatectomy with Dr. Ahlering.
